# Education Research: EEG Education in Child Neurology and Neurodevelopmental Disabilities Residencies: A Survey of US and Canadian Program Directors

**DOI:** 10.1212/NE9.0000000000200112

**Published:** 2024-01-05

**Authors:** Roohi Katyal, Irfan S. Sheikh, Aristides Hadjinicolaou, Christina Briscoe Abath, Elaine C. Wirrell, Shilpa B. Reddy, Sándor Beniczky, Fábio A. Nascimento

**Affiliations:** From the Department of Neurology (R.K.), Louisiana State University Health Shreveport, LA; Department of Neurology (I.S.S.), Massachusetts General Hospital, Harvard Medical School, Boston, MA; Department of Neurology (A.H.), CHU Sainte-Justine, Universite de Montreal, Canada; Department of Neurology (C.B.A.), Boston Children's Hospital, Harvard Medical School, Boston, MA; Department of Neurology (E.C.W.), Mayo Clinic Rochester, MN; Department of Neurology (S.B.R.), Vanderbilt University Medical Center Nashville, TN; Department of Clinical Neurophysiology (S.B.), Danish Epilepsy Center, Dianalund and Aarhus University Hospital; Department of Clinical Medicine (S.B.), Aarhus University, Denmark; and Department of Neurology (F.A.N.), Washington University School of Medicine St. Louis, MO.

## Abstract

**Background and Objectives:**

In the United States, many child neurologists (CNs) and neurodevelopmental disability (NDD) specialists who read EEGs in clinical practice had no additional EEG training other than what was received during residency. This practice highlights the importance of ensuring that CN/NDD residents achieve EEG competence before graduation. However, prior survey-based evidence showed that roughly a third of graduating CN residents in the United States do not feel confident interpreting EEGs independently. As part of a needs assessment, we conducted a descriptive study characterizing EEG practices in CN and NDD residency programs in the United States and Canada.

**Methods:**

A 30-question e-survey focused on characteristics of residency programs and their EEG teaching practices was sent to all 88 CN and NDD residency program directors listed in the Accreditation Council for Graduate Medical Education, Child Neurology Society, and Canadian Residency Matching Service websites.

**Results:**

Twenty-nine (n = 29/88; 33%) residency programs completed the survey, most of which were CN (90%), academic (90%), and located in the United States (83%). The mean number of weeks dedicated to EEG training required to graduate was 7.3 ± 4 (mean ± SD). EEG rotations involved the clinic/outpatient setting (83%), epilepsy monitoring unit (EMU) (76%), and inpatient setting (excluding EMU) (72%). During a 4-week EEG rotation, residents typically read 16–45 EEGs (62%). The most common methods of EEG teaching in CN/NDD programs were teaching during EEG rotation and yearly didactics. The mean number of EEGs read per rotation had a significant positive correlation with the average percentage of residents who reportedly achieve EEG competence by graduation (coefficient 0.461; *p* = 0.007). Barriers to EEG education were reported by 28% of the programs; the most common barrier identified was insufficient EEG exposure. Possible solutions were primarily related to increasing quality and quantity of EEG exposure. Almost two-thirds of programs reported not using objective measures to assess EEG competence.

**Discussion:**

Our results characterize resident EEG education in a third of CN/NDD residency programs in the United States and Canada. We suggest that residency leaderships consider standardization of EEG learning along with establishment and implementation of objective measures in training requirements and competence assessment.

## Introduction

In many countries, such as the United States and select European countries, neurologists without postresidency training in clinical neurophysiology (CNP)/EEG or epilepsy typically read EEGs in clinical practice.^1-5^ For instance, a survey-based study conducted by the American Academy of Neurology Member Research Subcommittee showed that 56.9% of respondents performed EEG making it the most common procedure performed by surveyed neurologists.^5^ Similarly, a significant number of European countries reported that general neurologists were either among the providers or the only providers who typically read EEGs in clinical practice.^3^ Survey data on the US child neurology workforce support that this practice also applies to child neurology (CN) and neurodevelopmental disability (NDD) specialists.^6^ This study showed that more than half of CN and NDD specialists who manage patients with epilepsy and read EEGs had no additional EEG training other than what was received during residency.^6^ It is, therefore, paramount to ensure that CN and NDD residents undergo optimal EEG training leading to proficiency by the time they graduate and start practicing independently.

Residency EEG training in the United States is guided by milestones proposed by the Accreditation Council for Graduate Medical Education (ACGME).^7^ Milestones range from level 1 to level 5 where level 4 is designed as a graduation goal and level 5 is designed to represent greater than expected achievements. Within the EEG subcompetency, it is recommended that CN residents should be able to describe general indications for an EEG (level 1); describe normal EEG features using correct terminology, including common artifacts, across the lifespan (level 2); describe patterns of status epilepticus, normal EEG variants, and common abnormalities across the lifespan (level 3); interpret common EEG abnormalities and create a report (level 4); and interpret uncommon EEG abnormalities (level 5).

Residency EEG training in Canada is guided by the Royal College of Physicians and Surgeons of Canada (RCPSC), which recommends that CN residents should gain competence in the physiologic basis of normal EEG and common EEG abnormalities; normal physiologic rhythms in wakefulness, drowsiness, and sleep; principal characteristics of neurophysiologic maturation in children; EEG indications and limitations; and recognition of common EEG abnormalities.

Despite the expectations concerning EEG learning during residency listed above, evidence suggests that about a third of graduating CN residents in the United States do not feel confident interpreting EEGs independently.^8^ These observations define an educational gap between the desired (CN residents systematically achieve EEG competence by graduation) and current (a significant portion of CN residents fail to become competent in EEG by graduation) educational practice. This is a descriptive study wherein we explore this educational gap by characterizing the CN/NDD resident EEG education practices in the United States and Canada as part of a needs assessment. This CN/NDD-specific data will add to the body of evidence concerning neurology trainee EEG education in the United States and abroad,^2-4,9^ thereby allowing our community to continue to improve EEG education that is curated and delivered to neurology trainees.

## Methods

We designed an online survey (eAppendix 1, links.lww.com/NE9/A57) focused on characteristics of CN and NDD residency programs and their EEG teaching systems. The survey was targeted at program directors of CN and NDD residency programs in the United States and Canada.

Key aspects of the survey development (including conducting a literature review, synthesizing the literature review, developing items, conducting expert validation, and conducting pilot testing) were in alignment with the guidance provided by the International Association for Medical Education Survey.^10^ First, we reviewed the literature in the field of trainee EEG education. We identified a published survey used to characterize EEG education practices in American adult neurology residency programs by collecting data from program directors.^4^ We also identified an adaptation of this survey that was used to characterize neurology resident EEG training in Europe.^3^ Four authors (R.K., I.S.S., A.H., F.A.N.) evaluated all items in these particular surveys and selected those that would be pertinent to CN/NDD resident EEG education in the United States and Canada. The latter items were further reviewed and, if necessary, adjusted, and additional questions were created to ensure that all salient aspects of American and Canadian CN/NDD EEG education were diligently addressed. Second, we conducted expert validation by consulting with 2 authors (S.B.R. and S.B.), during which process we discussed our preliminary survey items and addressed its representativeness, clarity, and relevance. Third, we conducted pilot testing by emailing the preliminary survey to 6 CN/NDD program directors. Of these, 1 anonymous CN/NDD program director completed the survey at this stage and provided us feedback. Finally, the survey was revised accordingly to contain 30 items comprising 20 multiple choice questions and 10 free-text questions.

The survey was hosted on Google Forms and sent through e-mail to program directors and program coordinators of all 88 CN and NDD residency programs listed on the ACGME, Child Neurology Society, and Canadian Residency Matching Service websites at the time of the study. Four biweekly reminders were sent to nonrespondent centers. Data collection was performed from February 2022 to April 2022. No financial compensation was offered.

Statistical analyses were performed using IBM (SPSS) Statistics version 28.0 software. Kendall's tau-b correlation was used to examine the relationship between select variables.

This study was conducted by the *Epileptic Disorders* Internship Program. The first 3 authors (R.K., I.S.S. and A.H.) were interns at the time of the study, and the senior authors (S.B. and F.A.N.) are Editors of the Internship Program.

### Standard Protocol Approvals, Registrations, and Patient Consents

This study was approved by the Institutional Review Board at the senior author's (S.B.) institution (EMN-2023-05622).

### Data Availability

Deidentified study data may be shared at the request of any qualified investigator.

## Results

Twenty-nine (n = 29/88; 33%) of the 88 CN and NDD residency program directors in the United States and Canada completed the survey; most programs were located in the United States (n = 24/29; 83%), and most were academic (n = 26/29; 90%). The 29 residency programs were hosted in a total of 26 institutions, all of which had a CN residency program (n = 26/29) and 3 of which also had an NDD residency program (n = 3/29). Programs' characteristics are summarized in Table 1.

**Table 1:** Comparison Between EEG Education Practices Among US and Canadian CN and NDD Residency Programs and US Adult Neurology Residency Programs

	Nascimento and Gavvala, 2021^a^	Present study
Residency programs included	Adult neurology (n = 47/161)	CN (n = 26/29) NDD (n = 3/29)
Response rate	29%	33%
Location of residency programs	United States	United States (83%) and Canada (17%)
Academic vs community based	Academic (89%) Community (6%) Mixed (4%)	Academic (90%) Community (0%) Mixed (10%)
EEG rotation(s) settings	Clinic/outpatient (70%) EMU/inpatient (91%)	Clinic/outpatient (83%) EMU (76%) Inpatient (excluding EMU) (72%)
Typical PGY during EEG rotation(s)	PGY1 (2%) PGY2 (50%) PGY3 (41%) PGY4 (7%)	PGY1 (3%) PGY2 (3%) PGY3 (76%) PGY4 (79%) PGY5 (48%) PGY6 (3%)
Primary methods of EEG teaching	Yearly didactics (95%) During EEG rotation (93%) During epilepsy clinic (66%) Bedside/inpatient rounds (52%) 1- to 2-mo didactics (30%) Other^b^ (16%) No formal didactics (0%)	Yearly didactics (93%) During EEG rotation (100%) During epilepsy clinic (86%) Bedside/inpatient rounds (86%) 1- to 2-mo didactics (17%) Overnight call (59%) Other^c^ (10%) No formal didactics (3%)
Main barriers to teaching EEG	No barriers (41%) Insufficient EEG exposure (32%) Ineffective didactics (11%) Other^d^ (34%)	No barriers (72%) Insufficient EEG exposure (10%) Ineffective didactics (7%) Insufficient didactics (3%) Limited teaching faculty/fellows (7%) Variation in depth/breadth of attending teaching (7%) Other^e^ (7%)
Mean number of EEG weeks required to graduate	6.8	7.3
Mean number of EEGs read during a typical (4-week) EEG rotation	0–10 (14%) 11–20 (20%) 21–30 (20%) 31–40 (11%) >40 (34%)	0–15 (0%) 16–30 (31%) 31–45 (31%) 46–60 (14%) >60 (24%)
Types of EEGs read during a typical (4-week) EEG rotation	N/A	Routine (100%) Prolonged routine (72%) Continuous (66%) EMU (55%) Ambulatory (38%)
Percentage of residents who met ACGME (if in the United States) EEG level 4 milestone or RCPSC (if in Canada) objectives of training by graduation	0%–20% (5%) 21%–40% (2%) 41%–60% (11%) 61%–80% (27%) 81%–100% (55%)	0%–20% (0%) 21%–40% (0%) 41%–60% (15%) 61%–80% (11%) 81%–100% (74%)
Use of objective measures to assess EEG competence	No (64%)	No (62%)

Abbreviations: CN = child neurology; N/A = not applicable; EMU = epilepsy monitoring unit; NDD = neurodevelopmental disability; PGY = postgraduate year.

a Survey respondents were adult neurology residency program directors in the United States.

b Overnight EEG reading on senior night float and using online EEG teaching platforms.

c EEG self-learning curriculum, online/e-learning, and bootcamp.

d Excessive inpatient workload and necessity for more residents, low/absent resident interest, and insufficient time to teach from faculty standpoint.

e No access to EEG software, limited time with EEG attending due to clinical duties.

Two of the 3 institutions with both a CN and NDD residency program opted to complete a single survey response for both CN and NDD programs given identical EEG education practices. The mean number of residents in each program—in all postgraduate years including preliminary years—varied significantly: 1–5 residents (n = 10/29), 6–10 residents (n = 12/29), 11–15 (n = 4/29), and >20 (n = 3/29). The mean number of residents who pursue postresidency fellowship training in CNP (with an EEG emphasis) and/or epilepsy was 0%–20% (n = 8/29), 21%–40% (n = 8/29), 41%–60% (n = 12/29), and 61%–100% (n = 1/29).

### EEG Rotation Characteristics

Programs vary in their requirements for the number of 1-month EEG rotations needed to graduate: zero (n = 2/29; 7%), 1 (n = 11/29; 38%), 2–3 (n = 15/29; 52%), and 4–5 (n = 1/29; 3%). The mean number of EEGs read (systematically reviewed from beginning to end) per resident during a typical (4-week) EEG rotation ranged from 0 to 15 (n = 0/29; 0%), 16 to 30 (n = 9/29; 31%), 31 to 45 (n = 9/29; 31%), 46 to 60 (n = 4/29; 14%), and >60 (n = 7/29; 24%). Detailed EEG rotation characteristics are summarized in Table 1.

### EEG Education: Resident Evaluation

Most programs (n = 26/28; 93%) reported not having a minimum number of EEGs residents are required to read and generate a preliminary report to fulfill EEG rotation requirements. Requirements for successful completion of an EEG rotation were collected by free-text responses. After data collection, the authors categorized these responses in 7 groups based on the primary nature of programs' requirements (Table 2).

**Table 2:** Requirements for Successful Completion of an EEG Rotation (Respondents, n = 29/29)

Responses (%)
1. Fulfillment of rotation objectives (55%)
2. Attendance only (10%)
3. Evaluation by preceptor (10%)
4. No specific requirement(s) (7%)
5. Examination(s) (7%)
6. Nonspecific responses (7%)
7. Number of EEGs read (3%)

The average percentage of residents meeting ACGME level 4 milestone (if in the United States) or RCPSC objectives of training for EEG (if in Canada) was 100% by the time of graduation in most programs (n = 19/27; 70%). In the remaining programs whose answers for this question were available, this figure decreased to 90% (n = 1/27; 4%), 80% (n = 2/27; 7%), 70% (n = 1/27; 4%), and 50% (n = 4/27; 15%). Less than half (n = 11/29; 38%) of programs reported using objective measures to assess resident EEG competence. In these programs, these measures were formal milestones (derived from ACGME, CNS, or not specified) (n = 7/11; 64%), program-specific EEG assessment criteria (n = 3/11; 27%), or examination(s) (n = 1/11; 9%).

There was a significant moderate^11^ positive correlation (coefficient 0.461; *p* = 0.007) between the average percentage of residents who reportedly achieve EEG competence by graduation (i.e., meet ACGME level 4 milestone or RCPSC objectives of EEG training if in the United States or Canada, respectively) and the mean number of EEGs read during a typical rotation. There was no significant correlation between the average percentage of residents who reportedly achieve EEG competence by graduation and (1) the mean number of EEG weeks required to graduate (coefficient −0.10, *p* = 0.954), (2) the mean number of residents enrolled in the program (in all postgraduate years) (coefficient 0.107, *p* = 0.539), or (3) the mean number of residents who pursue fellowship training in CNP (with an EEG emphasis) or epilepsy (coefficient 0.169, *p* = 0.333).

### EEG Education: Teaching Methods, Barriers, and Solutions

The most common primary mechanisms of EEG education were teaching during EEG rotation (n = 29/29; 100%) and yearly didactics (n = 27/29; 93%). Barriers to EEG education were reported by 28% (n = 8/29) of the programs. The most common barriers identified included insufficient EEG exposure (n = 3/29; 10%), ineffective didactics (n = 2/29; 7%), limited teaching faculty/fellows (n = 2/29; 7%), and variation in depth/breadth of attending teaching (n = 2/29; 7%). These data are summarized in Table 1. Possible solutions to overcoming these barriers and improving EEG education are listed in Table 3.

**Table 3:** Potential Solutions to Reported EEG Education Barriers (Respondents, n = 8/29, 28%)

1. Increasing the number of EEG rotations (n = 1/8)
2. Increasing the time residents spend reading EEGs with an attending (n = 1/8)
3. Protecting EEG reading time while in the EMU (n = 1/8)
4. Increasing autonomy for residents to take ownership of EEG reading and reporting (n = 2/8)
5. Integrating EEG education into core residency rotations (n = 1/8)
6. Providing residents with access to EEG software earlier in residency (n = 1/8)
7. Developing a centralized EEG curriculum based on content required for EEG certification examination (n = 1/8)

Abbreviation: EMU = epilepsy monitoring unit.

Each of the 8 programs provided only 1 potential solution.

## Discussion

Our study describes EEG education practices in one-third of CN and NDD residency programs in the United States and Canada. Programs included were mostly academic (90%), predominantly located in the United States (83%), and of varying sizes: from 1–5 to >20 residents per program. This study was undertaken as part of the *Epileptic Disorders* Internship Program.

The mean number of weeks dedicated to EEG training needed to graduate is 7.3 ± 4 (mean ± SD). Residents tend to complete EEG rotations during postgraduate years (PGYs) 3–5, and these rotations generally involve the outpatient/clinic setting (83%), epilepsy monitoring unit (EMU) (76%), and inpatient setting (excluding EMU) (72%). More than half of residents (62%) read between 16 and 45 EEGs during a typical (4-week) EEG rotation. The most common types of EEGs read are routine (100%) and prolonged routine EEGs (72%), continuous EEGs (66%), EMU EEGs (55%), and ambulatory EEGs (38%).

Adult neurology residents in the United States are required to undergo a comparable number of EEG training weeks (mean of 6.8) by graduation.^4^ Nonetheless, adult neurology trainees tend to read fewer EEGs per rotation: more than half of residents (54%) typically read 0–30 studies.^4^ Adult neurology residents' exposure to EEGs is slightly more centered in the EMU/inpatient setting,^4^ whereas CN/NDD residents' exposure is more balanced across outpatient and inpatient care environments. Finally, adult neurology residents generally undergo EEG training during PGY 2–3 compared with CN/NDD trainees who undergo training during PGY 3–5.

Neurology residents in Europe,^3^ particularly in European countries where general neurologists are either among the providers or the only providers who typically read EEGs (such as the United States), are required to undergo a mean of 9.2 weeks dedicated to EEG learning before graduation (excluding 1 country where training is continuous and 2 countries whose data are undetermined). Furthermore, in three-quarters of these European countries, residents read >40 EEGs during a typical EEG rotation.

Most CN/NDD programs (93%) have requirements for successful completion of EEG rotations. Nevertheless, these requirements are diverse and heterogenous, and range from attendance only (10%), “fulfillment of rotation objectives” (55%), to evaluations by preceptor(s) (10%). Furthermore, more than half of programs (62%) do not use objective measures to assess resident EEG competence. These characteristics are similar to EEG education practices in adult neurology residency programs in the United States.^4^ Conversely, a minority (31%) of European countries where general neurologists are either among the providers or the only providers who typically read EEGs does not use objective measures to assess EEG competence. In most CN/NDD (74%) and adult neurology (55%) programs, residents are reported to generally meet expected milestones by graduation.

On further statistical analysis, we identified a significant positive correlation (coefficient 0.461; *p* = 0.007) between the average percentage of CN/NDD residents who achieve EEG competence by graduation and the mean number of EEGs read per typical (4-week) EEG rotation. Interestingly, the former metric was not significantly correlated with the mean number of EEG weeks required to graduate, mean number of residents enrolled in the residency program, or the mean number of residents who pursue fellowship training in CNP (EEG emphasis) or epilepsy.

The most common methods of EEG teaching in CN/NDD programs consist of yearly didactics (93%) and teaching during EEG rotation (100%). Both methods were also the most used in adult neurology residencies in the US. CN/NDD residents, however, more frequently receive EEG education during epilepsy clinic (86% vs 66%) and bedside/inpatient rounds (86% vs 52%). In addition, learning EEG while on overnight call seems to occur often in CN/NDD programs (59%). In contrast to adult neurology programs, most CN/NDD residencies (72%) report no barriers to teaching EEG.

Our study has limitations. First, our sample of residency programs was skewed toward CN programs (vs NDD), academic centers (vs community-based), and those located in the United States (vs Canada). Second, our response rate of 33% imposes constraints as to generalizability of our findings on a binational level (the United States and Canada). Third, we evaluated program directors' perspectives without accounting for subjective or objective resident-based perspectives. Similarly, the average percentage of residents who meet EEG competence on graduation was assessed by program director report only, and not through objective measurement of residents' EEG skills. Fourth, we should be cautious when interpreting the significant positive correlation between the average percentage of residents achieving EEG competence by graduation and the mean number of EEGs read per typical (4-week) EEG rotation because correlation does not imply causation. It is likely that there are multiple other factors affecting these variables concurrently such as the heterogeneity of the study sample including varying degrees of resident autonomy, presence/absence of CNP/epilepsy fellows, level of faculty in teaching residents, and EEG rotation expectations.

In addition, we re-emphasize that the percentage of residents achieving EEG competence was assessed by program director report only; we did not obtain details on how this assessment was conducted and what factors were considered for such evaluation. Finally, we acknowledge that aspects of our survey development lacked key aspects of the recommendations proposed by the International Association for Medical Education Survey,^10^ namely the absence of distribution (a key component of expert validation) and the limited pilot testing.

In many countries across the world,^2,3^ including the United States, a significant portion of EEGs are read by neurologists without additional training in CNP or epilepsy. Particularly in the United States and specifically in the pediatric realm, it has been shown that more than half of providers who care for patients with epilepsy and read EEGs received EEG training only during residency. By contrast, in-residency EEG training may be improved as about a third of graduating CN residents in the United States reported not feeling confident in reading EEG independently.^8,9^

Consequently, it is critical to ensure that in-residency EEG education is rigorous and invariably effective, thus allowing graduates to be competent in EEG as they start practicing independently. We believe that achieving this milestone is strictly necessary in countries whose clinical EEG practice allows the interpretation of EEGs by neurologists without postresidency training in EEG—such as the United States. Ensuring that this milestone is universally met requires mandatory in-residency EEG training. Although the ideal number of EEG training weeks likely varies depending on multiple factors including the quality of EEG teaching, recent evidence suggests that residents should undergo more than 8 weeks (ideally more than 12 weeks) of EEG training to ensure minimal routine EEG competency.^12^ Our recommendations herein proposed are supported by the observation that EEG misinterpretation (predominantly related to EEG overreading) is the most common cause of epilepsy misdiagnosis.^13-15^ In other words, accurate and reliable EEG interpretation is imperative to provide optimal care to patients with seizures and epilepsy. Nevertheless, in scenarios where it is impractical to ensure that residents are competent in EEG by the time of graduation, programs may consider a different model where a more rigorous EEG training is required for those trainees who are expected to interpret EEGs in clinical practice after residency completion.

In countries where EEGs are read by neurologists with additional training in CNP or epilepsy, such as many European countries,^3^ in-residency EEG education continues to be of paramount importance. Residents and neurology graduates should still be competent in understanding the basics of EEG as well as its indications, common findings,^16^ standardized terminology,^17^ and—importantly—EEG reports. Mastering this knowledge is necessary for a provider to be able to indicate neurodiagnostics and understand EEG findings leading to optimal application of these data in patient care. We believe this applies to every neurology trainee even those who do not plan to pursue a career in CNP or epilepsy given the prevalence of people with seizures/epilepsy and the instrumental value of EEG in this patient population. Nevertheless, in these particular countries, resident EEG education may be tailored to the learning objectives listed above as opposed to ensuring that all trainees are able to accurately and reliably read EEGs in an independent fashion. Such tailored curricular framework may require a different approach to EEG learning and may potentially require a shorter duration of training.

We believe that the initial step toward improving EEG education in CN/NDD residency would be to address the most commonly reported barriers to EEG teaching by increasing the quantity and quality of EEG exposure. As to the former, our study results suggest that increasing the number of EEGs read per rotation may be particularly helpful toward ensuring EEG competence among residents. As to the latter, we would recommend the use of e-learning as an educational supplement^18-21^ as well as automated methods for teaching EEG interpretation^22^ and EEG pattern-specific training modules.^23^ Furthermore, we would highlight that tailoring EEG teaching depending on learners' prior EEG knowledge/exposure may be educationally beneficial; for instance, ensuring that residents have had hands-on EEG exposure before or during standard EEG lectures may generally maximize educational benefit.^21-23^ Table 4 presents the proposed future directions to improve EEG education in CN and NDD residency programs.

**Table 4:** Proposed Future Directions to Improve EEG Education in Child Neurology and Neurodevelopmental Disorders Residency Programs

1. Increase the quantity of EEG exposure (e.g., increase the number of EEGs read per rotation)
2. Improve the quality of EEG teaching (e.g., use e-learning as an educational supplement, use automated methods for teaching EEG interpretation, and use EEG pattern-specific training modules)
3. Adjust EEG teaching depending on learners' prior EEG knowledge/exposure (e.g., prioritize hands-on EEG exposure before or during standard EEG lectures)
4. Establish objective measures for training requirements
5. Establish objective measures for competence assessment (e.g., define minimum EEG competency milestones and assessment standards)

Finally, we believe that there is a need to establish objective measures concerning training requirements and competence assessment (e.g., defining minimum EEG competency milestones and assessment standards^16^) in combination with milestones published by national and international societies, such as the ACGME, RCPSC, and ILAE.

## Appendix Authors

**Table:**

Name	Location	Contribution
Roohi Katyal, MD	Department of Neurology, Louisiana State University Health Shreveport	Drafting/revision of the manuscript for content, including medical writing for content; study concept or design; analysis or interpretation of data
Irfan S. Sheikh, MD	Department of Neurology, Massachusetts General Hospital, Harvard Medical School	Drafting/revision of the manuscript for content, including medical writing for content; study concept or design; analysis or interpretation of data
Aristides Hadjinicolaou, MD	Department of Neurology, CHU Sainte-Justine, Universite de Montreal	Drafting/revision of the manuscript for content, including medical writing for content; major role in the acquisition of data; study concept or design
Christina Briscoe Abath, MD	Department of Neurology, Boston Children's Hospital, Harvard Medical School	Drafting/revision of the manuscript for content, including medical writing for content; analysis or interpretation of data
Elaine C. Wirrell, MD	Department of Neurology, Mayo Clinic	Drafting/revision of the manuscript for content, including medical writing for content; analysis or interpretation of data
Shilpa B. Reddy, MD	Department of Neurology, Vanderbilt University Medical Center	Drafting/revision of the manuscript for content, including medical writing for content; analysis or interpretation of data
Sándor Beniczky, MD, PhD	Department of Clinical Neurophysiology, Danish Epilepsy Center, Dianalund and Aarhus University Hospital; Department of Clinical Medicine, Aarhus University	Drafting/revision of the manuscript for content, including medical writing for content; study concept or design; analysis or interpretation of data
Fábio A. Nascimento, MD	Department of Neurology, Washington University School of Medicine	Drafting/revision of the manuscript for content, including medical writing for content; study concept or design; analysis or interpretation of data

## Glossary

ACGME: Accreditation Council for Graduate Medical Education
CN: child neurology
CNP: clinical neurophysiology
EMU: epilepsy monitoring unit
NDD: neurodevelopmental disability
PGY: postgraduate year
RCPSC: Royal College of Physicians and Surgeons of Canada
